# Prevalence and prognosis of hypoxia‐inducible factor‐2α (HIF‐2α) pathway gene mutations across advanced solid tumors

**DOI:** 10.1002/cam4.7358

**Published:** 2024-06-12

**Authors:** Wenjun Zhong, Jiemin Ma, Cai Chen, E. J. Dettman, Razvan Cristescu, Girish S. Naik, Fan Jin, Changxia Shao

**Affiliations:** ^1^ Merck & Co., Inc. Rahway New Jersey USA

**Keywords:** epidemiology, HIF‐2α, oncology, prevalence, prognosis

## Abstract

**Introduction:**

Hypoxia‐inducible factor‐2α (HIF‐2α) modulates the hypoxic response pathway in tumors; however, mutations in pathways (including SDHA, SDHB, SDHC, SDHD, FH, and VHL genes) that are suspected to activate HIF‐2α are poorly understood, with limited understanding of the prevalence and clinical prognosis.

**Methods:**

This retrospective observational study used a de‐identified nationwide (US‐based) clinico‐genomic database (CGDB) across 15 available tumor types.

**Results:**

Among the 9467 adult patients with advanced/metastatic solid tumors included in the analysis, any mutation at the above‐mentioned six genes was observed in 1.8% (95% CI: 1.5–2.1) of patients. The mutation prevalence ranged from 0.05% of SDHD to 0.93% of VHL. When further stratified by tumor type, the prevalence of gene mutation in each tumor type was well below 1%, except for VHL with 44% in renal cell carcinomas (RCC). Excluding RCC, the prevalence of any HIF‐2α gene mutations in the study population was 0.9% (95% CI: 0.8–1.2). The median overall survival (OS) from 1 and 2 L therapy among patients with any HIF‐2α gene mutation was 14.5 (95% CI: 11.5–24.2) and 9.3 (95% CI: 6.0–18.1) months, respectively, compared with 13.4 (95% CI: 12.9–13.9) and 9.8 (95% CI: 9.3–10.4) months among patients without HIF‐2α gene mutations.

**Discussion and Conclusions:**

The prevalence of HIF‐2α related gene mutations was generally low (<1%) across the 15 solid tumor types, except for VHL in RCC. No significant association between HIF‐2α gene mutation status and OS was identified among patients evaluated in this study.

## 1 INTRODUCTION

Intratumoral hypoxia plays a central role in cancer progression and is closely linked to poor patient prognosis and resistance to chemotherapy and radiation. Hypoxia‐inducible factor 2α (HIF‐2α) modulates the hypoxic response pathway in tumors, thus representing an attractive target for therapeutic intervention.^1^ Multiple genes are involved in the HIF pathway, including SDH (succinate dehydrogenase), VHL (von Hippel–Lindau), FH (fumarate hydratase), etc. HIF‐2α is activated through protein stabilization by loss of function mutations in VHL and certain members of the Krebs cycle, including SDH.^2^ SDH is a mitochondrial enzyme which participates in both the citric acid cycle and the electron transport chain; it is composed of six subunits encoded by SDHA, SDHB, SDHC, SDHD, SDHAF1, and SDHAF2.^3^ VHL gene is a tumor suppressor gene located on the short arm of chromosome 3, and von Hippel–Lindau syndrome is a familiar condition caused by pathogenic germline mutation of VHL gene.^4^ The FH gene encodes for fumarate hydratase, which helps cells in the body use oxygen. FH gene mutation may cause change in oxygen use in cells which may increase the growth of some cells, including abnormal cells and cancer cells. FH germline mutations were previously known to be responsible for the HRLCC syndrome (Hereditary Leiomyomatosis and Renal Cell Cancer) or Reed syndrome.^5^

Mutations in HIF‐2α pathways are suspected to activate HIF‐2α which is linked to cancer progression and prognosis. Increased accumulation of oncometabolites (from Krebs cycle)‐like succinate and fumarate (due to mutations in SDHx and FH genes [commonly seen in hereditary PPGL]) results in competitive inhibition of PHDs and thus HIF stabilization leading to a pseudohypoxic environment. On the contrary, mutations in the hypoxia signaling pathway (VHL, HIF‐2α, PHD1, and PHD2) can directly stabilize HIF‐2α, leading to upregulation of the VEGF and related pathways leading to tumor growth formation and progression.^2^, ^6^ However, there are limited data on the mutations of the genes included in HIF‐2α pathway and their relationship with clinical outcomes across tumor types from real‐world settings. Current available data are mainly among a few tumor types, such as pheochromocytoma and paraganglioma (PPGL), renal cell carcinomas (RCC), and gastrointestinal stromal tumors (GIST).^7^, ^8^, ^9^ There are few studies on prevalence of HIF‐2α–related gene mutations across multiple commonly diagnosed solid tumors. Therefore, in this study, we estimated prevalence of HIF‐2α–related gene mutations in 15 advanced/metastatic solid tumors and explored the prognosis on overall survival (OS) by mutation status.

## 2 MATERIALS AND METHODS

### 2.1 Data source

The data are from the nationwide de‐identified Flatiron Health and Foundation Medicine pan‐tumor Clinico‐Genomic Database (FH‐FMI CGDB) and originate from approximately 280 cancer clinics, which represent approximately 800 sites of care in the United States. A total of 15 tumor types were included in this CGDB database, including cancer of the bladder, breast, colorectal cancer (CRC), endometrial cancer, gastric cancer, head and neck (HN), hepatocellular carcinoma (HCC), malignant pleural mesothelioma (MPM), melanoma, non‐small cell lung cancer (NSCLC), ovaries, pancreas, prostate, RCC, and small cell lung carcinoma (SCLC).This dataset consists of both clinical (patient‐level structured and unstructured data) and genomic data with >300 cancer‐associated genes sequenced using the FoundationOne NGS assays (CDx) for over 100,000 patients.^10^ Retrospective longitudinal clinical data were derived from electronic health record (EHR) data and curated via technology‐enabled abstraction. These longitudinal clinical data were linked with FMI comprehensive genomic profiling data using a HIPAA‐compliant de‐identification process to create a continuously aggregating clinico‐genomic database. Data in the CGDB are updated on a quarterly basis to add new patients and to revise clinical data for existing patients and includes patients mainly from community practices. The de‐identified data were subject to obligations to prevent reidentification and protect patient confidentiality.

Lines of therapy administered to each patient in the cohort were defined by Flatiron Health business rules, which were established by expert oncology clinicians, and were derived from EHR data. Patient survival status was derived by combining multiple data sources and benchmarked against the National Death Index. The sources of data include EHR clinical data, commercial death data, and publicly available US mortality data from the Social Security Death Index.

### 2.2 Study population

The study population consisted of patients with one of the 15 solid tumor types available in the FH‐FMI CGDB: cancer of the bladder, breast, colorectal cancer (CRC), endometrial cancer, gastric cancer, head and neck (HN), hepatocellular carcinoma (HCC), malignant pleural mesothelioma (MPM), melanoma, non‐small cell lung cancer (NSCLC), ovaries, pancreas, prostate, RCC, and small cell lung carcinoma (SCLC). Patients were eligible if they were aged ≥18 years as of advanced/metastatic diagnosis date, had ≥2 documented clinic visits at a site in the Flatiron Health research network on or after January 1, 2011, diagnosed with advanced/metastatic solid tumors in 2018–2019, sequenced by the FoundationOne®CDx assay and had a non‐missing genomic loss of heterozygosity (gLOH) score. They were followed up till December 31, 2020, loss follow‐up or death, whichever was earlier.

### 2.3 Statistic method

In this study, HIF‐2α pathway gene mutations included deleterious mutations (nonsense mutations, frameshift mutations, and splice site mutations) and other mutation types reported as pathogenic or likely pathogenic variants in the ClinVar database. Prevalence of mutations of SDHA, SDHB, SDHC, SDHD, FH, and VHL genes was estimated with corresponding 95% confidence interval (CI). OS was defined as the time from initiation of first line (1 L) or second line (2 L) therapy to the date of death due to any cause or censor date. Patients without observed death were censored at the earlier date of loss of follow‐up (the last follow‐up date available in the dataset), or end of study follow‐up date (December 31, 2020).

OS with corresponding 95% CIs from the 1 L and the 2 L of therapy initiation were estimated with adjustment for delayed entry when the reported date of sequencing results was later than initiation of 1 L or 2 L therapy, stratified by gene mutation status (mutated vs. non‐mutated). Cox proportional hazards models were used to examine relative risks of survival from 1 L or 2 L therapy initiation by gene mutation status, adjusting for age, sex, race, practice type, and tumor type.

The IRB approval for the Flatiron Health Clinico‐Genomic dataset (CGDB) was obtained by Flatiron Health prior to the present study. The IRB of WCG Clinical (Princeton, NJ) gave ethical approval for the study protocol based on the Flatiron CGDB and included a waiver of informed consent (approval ID 420180044). All analyses were conducted by SAS 9.4.

## 3 RESULTS

**FIGURE 1:**
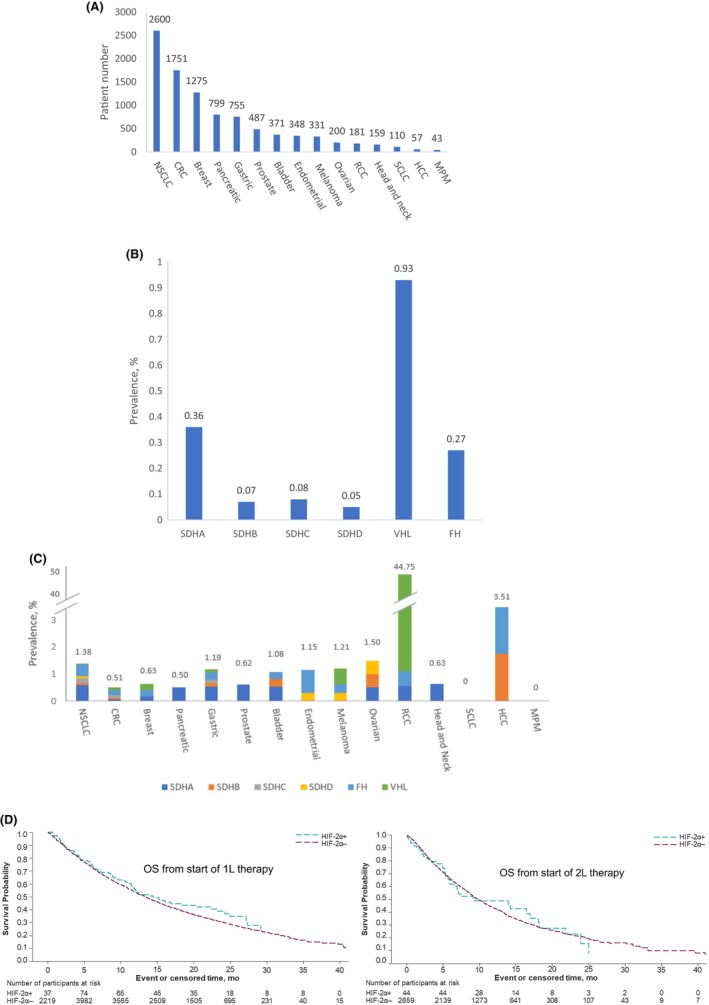
Distribution of tumor types (A), prevalence of HIF‐2α gene mutations (B, C) and OS by HIF‐2α gene mutation status (D). (A) Distribution of 15 tumor types in the study population; (B) Prevalence of HIF‐2α gene mutations (by each individual gene) across the 15 tumor types; (C) Prevalence of HIF‐2α gene mutations (by each individual gene) in each of the 15 tumor types; (D) OS by HIF‐2α gene mutation status, adjusted for age, sex, race, practice type, and tumor type; CRC: colorectal cancer; HCC: hepatocellular cancer; MPM: mesothelioma.; NSCLC: non‐small cell lung cancer; RCC: renal cell cancer; SCLC: small cell lung cancer.

In total, 9467 patients across 15 tumor types (Figure 1, panel A) were included in this study. The top five tumor types represented more than 75% of the patients, including non‐small cell lung (27%), colorectal (19%), breast (13%), pancreatic (8%), and gastric (8%) cancers. The median age was 66 years, 52% were women, 66% were White, and a little over half (54%) were stage IV disease at initial diagnosis. Overall, 2.2% of patients were MSI‐H; 18.9% were TMB‐H. The median follow‐up time was 13.1 months from 1 L therapy and was 9.5 months from 2 L therapy. The details of the demographic and clinical characteristics of the study population can be found in Appendix A: Tables A1 and A2.

Overall, the prevalence of any mutations of HIF‐2α‐ genes (i.e., SDHA, SDHB, SDHC, SDHD, FH, or VHL genes assessable in the FH‐FMI CGDB dataset) was observed in 1.8% (95% CI: 1.5%–2.1%) of patients; the mutation prevalence ranged from 0.05% of SDHD to 0.93% of VHL (Figure 1, panel B). When further stratified by tumor type, prevalence of gene mutation in each tumor type was also well below 1% to 2%, except for VHL with 44% in RCC (Figure 1, panel C). No concurrent mutations of any two HIF‐2α genes of the six genes were identified in this study.

Excluding RCC, the prevalence of any HIF‐2α gene mutations in the study population was 0.9% (95% CI: 0.8%–1.2%). No major differences in the prevalence were observed by age, race, gender, TMB status, or MSI‐H status (Table 1).

**TABLE 1:** Prevalence of HIF‐2α pathway mutation by patient characteristics.

	All patients			All patients excluding RCC		
	*N*	Mutated	Prevalence (95% CI)	*N*	Mutated	Prevalence (95% CI)
Overall	9467	168	1.77 (1.52, 2.06)	9286	87	0.94 (0.75, 1.15)
Gender						
Male	4573	98	2.14 (1.74, 2.61)	4452	40	0.90 (0.64, 1.22)
Female	4894	70	1.43 (1.12, 1.80)	4834	47	0.97 (0.72, 1.29)
Age, years						
<65	4362	79	1.81 (1.44, 2.25)	4270	37	0.87 (0.61, 1.19)
≥65	5105	89	1.74 (1.40, 2.14)	5016	50	1.00 (0.74, 1.31)
Race						
White	6212	111	1.79 (1.47, 2.15)	6101	57	0.93 (0.71, 1.21)
Black	686	10	1.46 (0.70, 2.66)	671	10	1.49 (0.72, 2.72)
Other	1814	33	1.82 (1.26, 2.55)	1776	15	0.84 (0.47, 1.39)
Unknown	755	14	1.85 (1.02, 3.09)	738	5	0.68 (0.22, 1.57)
TMB						
TMB <10	7676	137	1.78 (1.50, 2.11)	7498	57	0.76 (0.58, 0.98)
TMB ≥10	1790	31	1.73 (1.18, 2.45)	1787	30	1.68 (1.14, 2.39)
Unknown	1	0	0 (n.a.)	1	0	0 (n.a.)
MSI						
MSI‐H	213	8	3.76 (1.64, 7.27)	213	8	3.76 (1.64, 7.27)
Non‐MSI‐H	8365	144	1.72 (1.45, 2.02)	8203	73	0.89 (0.70, 1.12)
Unknown	889	16	1.80 (1.03, 2.91)	870	6	0.69 (0.25, 1.50)
Stage at initial diagnosis (I‐IV)						
Stage I	634	7	1.10 (0.45, 2.26)	634	7	1.10 (0.45, 2.26)
Stage II	1151	8	0.70 (0.30, 1.36)	1151	8	0.70 (0.30, 1.36)
Stage III	1749	12	0.69 (0.36, 1.20)	1749	12	0.69 (0.36, 1.20)
Stage IV	5135	87	1.69 (1.36, 2.09)	5047	51	1.01 (0.75, 1.33)
Other/unknown	798	54	6.77 (5.12, 8.74)	705	9	1.28 (0.59, 2.41)

The median OS from 1 L and 2 L therapy among patients with any HIF‐2α gene mutation was 14.5 (95% CI: 11.5–24.2) and 9.3 (95% CI: 6.0–18.1) months, respectively, compared to 13.4 (95% CI: 12.9–13.9) and 9.8 (95% CI: 9.3–10.4) months among patients without HIF‐2α gene mutations (Figure 1, panel D). The crude and adjusted hazard ratios (HRs) for OS from 1 L therapy initiation for patients with any HIF‐2α gene mutations versus HIF‐2α gene mutation–negative patients was 0.9 (95% CI: 0.7–1.1) and 1.1 (95% CI: 0.8–1.4), respectively. The crude and adjusted HR of OS from 2 L therapy initiation was 1.0 (95% CI: 0.7–1.4) and 1.3 (95% CI: 0.9–1.8) for patients with any HIF‐2α gene mutations versus HIF‐2α gene mutation–negative patients, respectively.

Any association between each individual gene and OS was also explored. No significant association was found between each individual gene and OS from 1 L therapy. For the association between each individual gene and OS from 2 L therapy, only SDHA mutation was associated with a shorter OS (4.2 vs. 9.9 months, adjusted HR 2.83 (1.46–5.47)), no other significant associations were identified for the other five HIF‐2α pathway genes. (Table 2).

**TABLE 2:** OS from 1 L therapy or 2 L therapy by HIF‐2α gene mutation status.

Index date for OS	Gene	Mutated	Non‐mutated	aHR^a^ (95% CI)
1 L	Any HIF‐2α gene	60/146	3673/7730	1.05 (0.80; 1.40)
1 L	SDHA	14/28	3719/7848	1.10 (0.65, 1.86)
1 L	SDHB	3/7	3730/7869	1.40 (0.44, 4.40)
1 L	SDHC	1/8	3732/7868	0.16 (0.02, 1.14)
1 L	SDHD	2/4	3731/7872	1.42 (0.35, 5.73)
1 L	VHL	27/76	3706/7800	1.02 (0.63, 1.64)
1 L	FH	13/23	3720/7853	1.58 (0.92, 2.74)
2 L	Any HIF‐2α gene	36/77	2041/4288	1.28 (0.88, 1.84)
2 L	SDHA	9/15	2068/4350	2.83 (1.46, 5.47)
2 L	SDHB	0/1	2077/4364	NE
2 L	SDHC	1/5	2076/4360	0.17 (0.02, 1.21)
2 L	SDHD	0/1	2077/4364	NE
2 L	VHL	19/42	2058/4323	1.61 (0.86, 3.00)
2 L	FH	7/13	2070/4352	1.50 (0.71, 3.15)

^a^ Adjusted for age, sex, race, practice type, and tumor type.

## 4 DISCUSSION

To our knowledge, this is the first report on HIF‐2α gene mutations across 15 solid tumor types and the first to assess their prognosis. The prevalence of any HIF‐2α gene mutation in the six genes (SDHA, SDHB, SDHC, SDHB, FH, and VHL) was generally low (<1%) across the 15 tumor types, except for VHL mutation among RCC (which was 44%). The prevalence does not seem to differ by age, gender, race, or MSI‐H/TMB‐H status. Among all patients, “other/unknown” disease stage had higher prevalence than other disease stages, but the difference was no longer prominent after excluding RCC. This is likely because many of these “other/unknown” cases were RCC cases which harbor higher prevalence of VHL gene mutation.

The relatively high prevalence of VHL gene mutation in RCC has been previously reported, in a range of 40% to 70%.^8^ It is also believed that VHL gene mutation is not associated with the pathologic features and survival in RCC patients.^11^ Our study finding on the prevalence of VHL gene mutation in RCC is consistent with the literature and we also did not find association between VHL gene mutation with OS across all tumor types. VHL mutation has also been reported in other tumor types: about 30%–40% in hemangioblastomas, 2%–11% in pheochromocytomas, 5%–15% in endolymphatic sac tumor (ELST), and 1% in pancreatic neuroendocrine tumor (PNET).^12^ However, none of these rare tumors were included in our study, thus we were not able to compare the results.

Current available studies on SDH genes are mainly focused on PPGL. A meta‐analysis of PPGL studies reported that the prevalence of SDHA, SDHB, SDHC, and SDHD were 1.1% (95% CI: 0.6–2.2), 8.3% (95% CI: 6.4–10.5), 0.4% (95% CI: 0.1–1.2), and 1.3% (95% CI: 0.6–2.4), respectively. In metastatic PPGL, SDHB mutation prevalence could be as high as 35%.^7^ The association of SDHB mutation with PPGL patient's survival, however, was inconsistently reported in the literature.^13^, ^14^, ^15^ Our study did not include PPGL, and no significant association between SDH mutations and OS was found, except that SDHA mutation was associated with shorter survival from 2 L therapy initiation. However, this finding should be interpreted cautiously because the sample size is small (only 15 patients with mutated SDHA) and it is subject to residual confounding due to lack of some potentially important confounding adjustment (such as adjustment of treatments, comorbidities, etc.). To our knowledge, there have been no prior reports identifying an association between SDHA mutation and overall survival. Further studies are needed to investigate whether SDHA is associated with prognosis.

Besides being known to be responsible for HRLCC syndrome or Reed syndrome, the FH gene recently was found to be involved in PPGL, with mutation prevalence about 0.83%.^16^ One study of FH deficient RCC patients suggested these patients had long OS from 1 L systemic treatment; however, no comparator arm was included in this study.^17^ Apart from the above, there are few data on FH gene mutation prevalence across tumors. Our study provided such data and found that the prevalence of FH mutation was generally below 1% across the 15 tumor types and was not associated with OS.

The strengths of this study included a relatively large sample size, and the same comprehensive genetic testing consistently applied to all the study patients. The study also has limitations. First, due to data availability, the study was limited to HIF‐2α genes covered by F1CDx panel and patients with a non‐missing gLOH score, and it is unknown whether gLOH evaluability is related to HIF‐2α gene mutations. Also, patients from this clinical genomic database may not be representative of all cancer patients in United States. Thus, the derived prevalence from the present study may not be generalizable to general cancer patients. Second, depending on tissue collection time, the samples tested in this study may not always reflect the biomarker status of the advanced/metastatic tumor stage, if the genetic profile changes by disease stage or by treatment regimen. Nonetheless, more than half of the patients were stage IV at initial diagnosis, and this study reported meaningful data on HIF‐2α gene mutations among patients with advanced/metastatic cancers where there is high unmet medical need. Finally, due to the rarity of HIF‐2α gene mutation, it is not feasible to conduct meaningful survival analysis by tumor types.

In conclusion, our study found the prevalence of HIF‐2α‐related gene mutations was generally low (<1%) across 15 solid tumor types, except for VHL in RCC. No significant association between HIF‐2α gene mutation status and OS was identified among these patients with advanced/metastatic solid tumors.

## AUTHOR CONTRIBUTIONS

**Wenjun Zhong:** Conceptualization (equal); formal analysis (equal); investigation (equal); methodology (equal); project administration (equal); validation (equal); visualization (equal); writing – original draft (equal); writing – review and editing (equal). **Jiemin Ma:** Conceptualization (equal); data curation (equal); formal analysis (equal); investigation (equal); methodology (equal); software (equal); validation (equal); visualization (equal); writing – original draft (equal); writing – review and editing (equal). **Cai Chen:** Conceptualization (equal); data curation (equal); formal analysis (equal); investigation (equal); methodology (equal); software (equal); writing – review and editing (equal). **E. J. Dettman:** Conceptualization (equal); data curation (equal); formal analysis (equal); investigation (equal); methodology (equal); visualization (equal); writing – review and editing (equal). **Razvan Cristescu:** Conceptualization (equal); data curation (equal); formal analysis (equal); investigation (equal); methodology (equal); writing – review and editing (equal). **Girish S. Naik:** Conceptualization (equal); formal analysis (equal); investigation (equal); methodology (equal); writing – review and editing (equal). **Fan Jin:** Conceptualization (equal); formal analysis (equal); investigation (equal); methodology (equal); writing – review and editing (equal). **Changxia Shao:** Conceptualization (equal); data curation (equal); formal analysis (equal); investigation (equal); methodology (equal); resources (equal); supervision (equal); validation (equal); visualization (equal); writing – original draft (equal); writing – review and editing (equal).

## FUNDING INFORMATION

Funding for this research was provided by Merck Sharp & Dohme LLC, a subsidiary of Merck & Co., Inc., Rahway, NJ, USA.

## CONFLICT OF INTEREST STATEMENT

All the authors are Merck Sharp & Dohme LLC, a subsidiary of Merck & Co., Inc., Rahway, NJ, USA employees and may hold stock and stock options of Merck & Co., Inc., Rahway, NJ, USA.

## Data Availability

The data that support the findings of this study originated by Flatiron Health, Inc., and Foundation Medicine, Inc. These de‐identified data may be made available upon request, and are subject to a license agreement with Flatiron Health and Foundation Medicine; interested researchers should contact <cgdb-fmi@flatiron.com> and <publicationsdataaccess@flatiron.com> to determine licensing terms.

## APPENDIX A

**TABLE A1:** Baseline demographic and clinical characteristics of study population.

	All	HIF‐2α mutated	HIF‐2α non‐mutated
*N* (%)	9467 (100.0)	168 (1.8)	9299 (98.2)
Gender			
Male	4573 (48.3)	98 (58.3)	4475 (48.1)
Female	4894 (51.7)	70 (41.7)	4824 (51.9)
Age, years			
Mean (SD)	64.5 (11.6)	65.0 (10.9)	64.5 (11.6)
Median (Q1, Q3)	66.0 (57.0, 73.0)	65.0 (58.0, 74.0)	66.0 (57.0, 73.0)
<65	4362 (46.1)	79 (47.0)	4283 (46.1)
≥65	5105 (53.9)	89 (53.0)	5016 (53.9)
Race			
White	6212 (65.6)	111 (66.1)	6101 (65.6)
Black	686 (7.2)	10 (6.0)	676 (7.3)
Asian	194 (2.0)	2 (1.2)	192 (2.1)
Other	1620 (17.1)	31 (18.5)	1589 (17.1)
Unknown	755 (8.0)	14 (8.3)	741 (8.0)
Practice type			
Academic	1113 (11.8)	25 (14.9)	1088 (11.7)
Community	8354 (88.2)	143 (85.1)	8211 (88.3)
Stage at initial diagnosis			
Stage I	634 (6.7)	7 (4.2)	627 (6.7)
Stage II	1151 (12.2)	8 (4.8)	1143 (12.3)
Stage III	1749 (18.5)	12 (7.1)	1737 (18.7)
Stage IV	5135 (54.2)	87 (51.8)	5048 (54.3)
Other/Unknown	798 (8.4)	54 (32.1)	744 (8.0)
TMB			
TMB <10	7676 (81.1)	137 (81.5)	7539 (81.1)
TMB ≥10	1790 (18.9)	31 (18.5)	1759 (18.9)
Unknown	1 (0.0)	0 (0.0)	1 (0.0)
MSI			
MSI‐H	213 (2.2)	8 (4.8)	205 (2.2)
Non‐MSI‐H	8365 (88.4)	144 (85.7)	8221 (88.4)
Unknown	889 (9.4)	16 (9.5)	873 (9.4)
Follow‐up from 1 L, month			
Subjects with data, n	7876	146	7730
Mean (SD)	14.0 (9.6)	13.6 (8.8)	14.0 (9.6)
Median (IQR)	13.1 (12.9)	12.1 (14.4)	13.1 (12.8)
Follow‐up from 2 L, month			
Subjects with data, n	4365	77	4288
Mean (SD)	9.5 (8.41)	9.9 (7.45)	9.5 (8.43)
Median (IQR)	7.8 (9.6)	7.5 (9.1)	7.8 (9.6)

**TABLE A2:** Overall survival from 1 L at 6 months, 12 months by HIF‐2α mutation status.

	OS (mo, 95% CI)			
	*N*	OS (mo, 95% CI)	Prob of alive at 6 m (95% CI)	Prob of alive at 12 m (95% CI)
Any HIF‐2α gene mutations +	146	14.49 (11.50, 24.25)	0.75 (0.66, 0.84)	0.55 (0.46, 0.66)
Any HIF‐2α gene mutations −	7730	13.37 (12.88, 13.87)	0.73 (0.72, 0.74)	0.53 (0.52, 0.55)
